# Brain Structure and Function: the first 15 years—a retrospective

**DOI:** 10.1007/s00429-021-02362-0

**Published:** 2021-08-10

**Authors:** Laszlo Zaborszky

**Affiliations:** Center for Molecular and Behavioral Neuroscience, Rutgers University, 197 University Avenue, Newark, NJ 07102 USA

## History

The idea of a new journal, entitled *Brain Structure and Function* (BSAF), was conceived 15 years ago; in the Summer of 2006 in the office of Professor Karl Zilles,^1^ then Chair of the Anatomy Department in Düsseldorf. Through visits in Düsseldorf and later in Jülich, where Dr. Zilles established a Neuroscience Research Institute in the former nuclear research facility, we had many discussions about how a common anatomical framework, unconstrained by a single technique or level of analysis, can provide an opportunity to integrate the massive amount of data from genes and molecules to behavior and cognition to explore structure–function relationship from a new perspective.

When we started BSAF, most journals had responded to the need for specialization, dealing with various aspects of research, such as a brain region (*Cerebral Cortex, Hippocampus*), method (*Journal of Neuroscience Methods*), aspect of analysis (*Behavioral and Brain Sciences*) or a function (*Learning & Memory*). While these individual journals reflected the enormous growth of neuroscientific information, since the mid-1970s, it had also become obvious that none of the specific disciplines alone could fully explain how the brain works. We hoped that the expanding computational approach in neuroanatomical data acquisition, together with a combination of large-scale electrode recordings and imaging studies in behaving animals, would ultimately allow us to identify the key principles that relate circuitry architecture to the activity patterns of functional cell assemblies within specific cognitive operations at cellular and millisecond resolution. Furthermore, we predicted that the expanding neuroinformatics tools would allow for the complete digital representation of both cellular morphology and system-level anatomy of the entire rat brain and perhaps the primate brain within a decade (see Preface to Neuroanatomical Tract Tracing 3: Zaborszky et al. 2006; Nadasdy et al. 2006; Zaborszky and Zilles 2007). As it turned out, this was an illusion, similar to the plan that the human brain (Blue Brain Project & the Human Brain Project) could be simulated at cellular resolution within 10 years in a supercomputer (see the Epistemological Lessons Collection at eNeuron.org Bernard 2021; Destexhe 2021; Fairhall 2021; Fregnac 2021; Jirsa 2021). Nevertheless, we held our inaugural meeting with the newly elected board members in Atlanta at the Society for Neuroscience Meeting. Andrea Pillman,^2^ from Springer, helped to organize this meeting, and officially announced that Springer will re-launch *Anatomy and Embryology*, a journal that had been edited by Karl for many years, with new aims and scope^3^.

Although there were some concerns by a few members that we would face enormous difficulties due to the increased number of journals, the majority of scientists thought that BSAF would fill an important niche for system-level neuroanatomy papers devoted to integrative structure–function analysis. Initially, we had only a handful of Associate Editors, and thus, Zilles and I had to function not only as reviewing editors, but also in handling the difficult task of finding reviewers. Nevertheless, we raised the Impact Factor from a mere 2.4 in 2008 to 7.8 in 2012, thanks to several special issues, including “Transgenic Modeling of Neurodegenerative Diseases” (Guest editors: Patrick Hof and G.A. Elder 214/2–3, March 2010) and the Insula (Guest editor: Craig Budd; 214/5–6, June 2009). We also published a special issue dedicated to Lennart Heimer, who was a pioneer in neuroanatomical tract-tracing and the conceptualization of the new organization of the basal forebrain (213/1–2, September 2008; Heimer et al. 1991; Haber et al. 2008). We managed to increase the total page count of BSAF from 520 pages in the first volume^4^ in 2008 to over 4750 pages in 2016. Unfortunately, the increase in volume and increase of predatory journals, among other factors, led to the erosion of our place in the top quartile of neuroscience journals with an Impact Factor of 7.8 for 2012, though BSAF is still first among 20 Anatomy and Morphology journals. Edmund Rolls’ review on the cingulate gyrus (Rolls 2019) received 7444 access requests! It is needless to mention that in the early years even articles showing a single technique with two authors, such as the one investigating inputs to the prefrontal cortex (Hoover and Vertes 2007), achieved high numbers of citations. More recently, the number of authors listed per article has dramatically increased due to the multitude of techniques applied.

The popularity of the journal both for authors and readers may be due in part that, in addition to generous free color printing and special issues, we developed several columns—so-called “Letters to the Editor”, including *Brain Mythology* which fosters discussion of widely accepted but unproven dogmas (Hilgetag and Barbas 2009), and *Practical Guides* (e.g., Destrieux et al. 2017) that support the transfer of established knowledge from a specialized area of research to a broader readership. We initiated *Technical Notes*/*Methods* papers, and ‘*Letters to the Editor*’ also served to provide commentary on published papers with controversial findings (see Kocsis et al. 2018; Ruddy 2017). This last column is published as back-to-back papers with the response from the authors of the original paper.

In 1993, Francis Crick and Ted Jones published an influential paper in *Nature* noting the backwardness of human neuroanatomy (Crick and Jones 1993; see also Rushmore et al. 2020). Along this line, a mission of BSAF was to publish primate anatomy studies concerning characterization of structures and the investigation of their connections with special reference to cortical cyto-, myeloarchitectonics, receptor and genoarchitectonics, including comparative studies. Another pillar of BSAF is cellular neuroscience, and over the years we published many excellent papers related to synaptic architecture, physiological and morphological identification of single cells in the cortex, hippocampus, and circuit analysis in subcortical regions, such as basal ganglia, hypothalamus and the neuromodulatory systems.

I am revisiting papers published between 2007 and 2021 in BSAF, focusing on primate anatomy. These papers are a sample of a sample, representing the breadth and depths of research publication, not necessarily considering impact or citation. Since according to our intention, BSAF will publish without constraints with respect to methods or level of analysis, papers during this period covered the entire neuroscience enterprise^5^ from cellular resolution transcriptome (e.g., Wagner et al. 2016) to the frequency architecture of the human brain network (e.g., Rojkova et al. 2016). Between 2007 and 2020, BSAF published 1816 papers; however, due to restrictions on self-citation to avoid biasing impact factor, many excellent papers had to be removed from citation. References in this paper were done without a search engine, reflecting the narrow view of the ‘retiring’ Editor-in-Chief.

## Primate anatomy

### Atlasing and cytoarchitecture

Brain atlases are widely used in experimental neuroscience as tools for locating and targeting specific brain structures. Delineated structures in a given atlas, however, are often difficult to interpret and in the early 2000s efforts focused on interfacing atlases with database systems that supply additional information using hierarchically organized vocabularies (ontologies) (Martin and Bowden 2000; Koslow and Subramanian 2005; Nadasdy et al. 2006; Bezgin et al. 2009). In the twentieth century, Oscar and Cecile Vogt, Brodmann, von Economo, Flechsig, Baily and von Bonin, Sarkisov and others divided the human cortex into various areas, using cyto-and myeloarchitetonics. Similarly, cytoarchitectonics of monkey cortex were published (Preuss and Goldman-Rakic 1991; Barbas and Pandya 1987; Luppino et al. 1991). Rudolf Nieuwenhuys re-evaluated the myeloarchitectonic studies of the Vogt-Vogt school in the light of functional neuroimaging data (Nieuwenhuys et al. 2015). However, these maps have many shortcomings due to (i) lack of observer-independent quantification; (ii) lack of data on the intrasulcular surface that makes up 2/3 of the cortical surface; (iii) lack of knowledge on interindividual differences; and (iv) lack of conversion from 2 to 3D. To solve this problem, Zilles and his school invented two ingenious techniques: first, they established a method enabling statistical validation of the position of cortical borders, and second, donated brains were perfused, in situ imaged by T1-weighted MRI scanning, and then processed with traditional histologic techniques. Cortical regions were identified using the quantitative technique, delineated on 2D images of the sections, and overlaid in a reference brain: this statistically testable probabilistic 3D mapping became a standard technique in many laboratories around the world to correlate microstructural details with functional data.

From the Zilles’ school, a detailed multimodal mapping and analysis of the human hippocampus was published by the group of Nicola Palomero-Gallagher^6^ (Palomero-Gallagher et al. 2020); also of the human amygdala by Kedo et al., (2018). Other architectonic papers were produced from Zilles’s^7^ school lead by Katrin Amunts^8^; e.g., human supplementary and pre-supplementary motor areas (Ruan et al. 2018). The correlation of cytoarchitetonic data with receptor architectonic and genomic (transcriptomic) analysis to decode chains from genes to cognition and cognitive disorders has been a major advance; for example, the role for MAOA and TAC1 in the limbic-cortical network (Bludau et al. 2018). BSAF was among the first to publish the cyto-and chemo-archirtectonic atlas of the mouse prefrontal cortex (Van De Werd and Uylings 2014), which served an important comparative purpose in the parcellation of the human prefrontal cortex (Samara et al. 2017). Eickhoff^9^ and their coworkers (Zhang et al. 2015) used resting state fMRI data for brain parcellation of the medial PFC by constructing a sparse similarity graph based on the sparse representation coefficients of each seed voxel and then used spectral clustering to identify distinct modules. An atlas of the basal ganglia and thalamus of the macaque in stereotaxic coordinates was published by Lanciego and Vazquez (2012). 3D stereotaxic localization of the cerebellothalamic and pallidothalamic tract in the human brain, crucial in stereotaxic functional neurosurgery, was published by Gallay et al. (2008).

### MRI tractography

The introduction of Diffusion Tensor Imaging (DTI) tractography caused a revival of older postmortem anatomical fiber dissection techniques, such as the Klinger’s anatomical dissection and connectivity in non-human primate, is extensively used to validate tractography in humans (Mars et al. 2016). The advent of DTI allowed researchers to move human anatomy from being descriptive to detailing connections that can be interpreted more in terms of functions; however, ‘MRI tractography can be highly anatomically accurate—if we know where white matter pathways start, where they end and where they do not go’: the title of a paper discussing the limited sensitivity and specificity of diffusion MRI tractography (Schilling et al. 2020). MR histology is bridging the gap between MR and retroviral injection by acquiring a mesoscale connectome of the whole mouse (Wang et al. 2018). With the increasing popularity of small animal MRI techniques, there is considerable interest in methods that rapidly perform segmentation of neurological structures for quantitative comparisons. Gyengesi et al. (2014) used DTI tractography for the segmentation of major white matter tracts and compared this with various histology staining in the rat brain.

### Comparative neuroanatomy

For comparative purposes, BSAF published anatomy works in *birds*, *dolphin*, *ferret*, *marmoset*, mongolian *gerbil*, the pale spear-nosed *bat, tree shrew* and the African *elephant*. For instance, studies compared the anatomy of the socio-emotional circuit in bonobo and chimpanzee (Issa et al. 2019); examined neuron numbers in amygdala (Lew et al. 2018), and detailed the connections of dolphins (Wright et al. 2018).

### Cognitive and neurodevelopment

The classical theory of cortical systematic variation has been independently described in reptiles, monotremes, marsupials, and placental mammals, including primates, suggesting a common bauplan in the evolution of the cortex. The Structural Model by Barbas and her colleagues (Garcia-Cabezas et al. 2019) is a platform for advancing testable hypotheses linking connections, plasticity, development, evolution, variability of diseases across species, including humans**.** BSAF also published studies on prenatal cortical development including a framework to align preterm, infant, and adult human brain images acquired in vivo and postmortem (Lebenberg et al. 2018).

Patients with neuropsychiatric disorders often express limbic circuit abnormalities and deficits in information processing. While these disorders appear to have diverse etiologies, their common features suggest neurodevelopmental origins. Neurodevelopment is a prolonged process of diverse events including neurogenesis/apoptosis, axon pathfinding, synaptogenesis, and pruning, to name a few. The precise timing of the neurodevelopmental insult to these processes likely determines the resulting functionāl outcome. Fenton and his coworkers (O’Reilly et al. 2018) used the epilepsy and schizophrenia-related gestational day 17 methylazoxymethanol acetate model to examine the impact of this timed neurodevelopmental insult on principal cell morphology and synaptic network function of the dorsal hippocampus circuit. These data indicate that adverse global exposure during gestation can produce specific alterations in neural circuits that underlie cognitive abilities.

## Brain organization

In the light of burgeoning papers using the Connectome database, the understanding of the organization of brain networks is an important topic. Graph theory, a field of mathematics, deals with complex technical, social or biological systems, initiated by Erdos and Renyi (1960) describing the ‘random network’. However, in a random network most nodes^10^ have roughly the same number of links, equal to the network’s average degree (Poisson degree) distribution and this organization does not explain the behavior of most real networks. Watts and Strogatz (1998) described the mathematical properties of the ‘small-world’ network that is highly clustered, such as a regular graph, yet with small path length, such as a random graph. A year later, Barabasi and Albert (1999) published the ‘scale-free’ network that is characterized by a power-law degree distribution, meaning most nodes have only few connections, but a few nodes have many. The small-world network is between a regular and a random network, with many nearest-neighbor and few distant connections. Identifying the characteristics of networks is important, since they can statistically be evaluated and compared across species. The topology defines dynamic features, including adaptation, robustness and synchronization of widely separated neurons. In disease models, one can test which nodes can be eliminated without major disturbance, but removal of the richly connected hubs would lead to collapsing the system (Kaiser et al., 2007).

Hilgetag and Goulas (2016), in a *Brain Mythology* column of BSAF, debate whether the cortex is really a small-world network and emphasize the importance of scales. For example, synaptic connectivity within local neuronal populations might form small-world or random networks at the global scale; however, the cortex resembles large-world networks with hierarchical modularity and with scale-free topology, assembled from smaller subnetworks. The topological organization of cortico-cortical connections is a hot topic: the distance and ‘similar is connected to similar’ [architectonic] principles, originally formulated in the macaque monkey, has been confirmed in cat, mouse, rat, and human (van den Heuvel et al. 2016; Goulas et al. 2017). Despite certain similarities in the hierarchical cortical organization among mammalian species, Watakabe and Hirokawa (2018), using the Allen Mouse Brain Connectivity Atlas, describe differences in gray/white matter segregation of axonal projections between rodents and primates.

Single-axon tracing studies significantly contribute to organizational principles that are critical to interpreting neural dynamics. Rockland (2020), in her study, describes the organizational principles of thalamo-cortical and cortico-cortical connections in primates, including differences in laminar specificity between feedforward and feedback connections, important in visual processing. Single-axon tracing methods have been also used in the rat hippocampus from the Buzsaki laboratory, reconstructing the axonal arborization of a CA3 pyramidal neuron published in the first issue of BSAF (Wittner et al. 2007). Single-axon tracing in the rat barrel cortex suggests that axons radiate in all directions for over 3.5 mm, which may contribute to multimodal cortical responses and various forms of cortical plasticity (Johnson and Frostig 2016). Duque et al. (2007) describes the morphology of electrophysiologically identified cholinergic and NPY neurons in the basal forebrain.

## Structure–function approaches in animal experiments and in human imaging studies

Links between anatomy and function can be investigated at various scales, though these links are often established through correlational analysis, as it happens in most of the human imaging studies. Here we give a few examples from the literature, including papers published in BSAF, where this correlational approach produced remarkable progress. At the end of this section, we list a few examples from animal research, where anatomy can be translated to function due to the sophisticated combination of methods in un-anesthetized animals. BSAF published in 2009 a special issue entitled “Imaging the relationship between structure, function and behavior” edited by Heidi Johansen-Berg. As she explains in the introduction (Johansen-Berg 2009), brain anatomy and functional responses are inextricably linked and together determine our behavioral output.

Systematic, spontaneous, low-frequency fluctuation was first demonstrated by Biswal et al. (1995) using fMRI in the motor cortex during resting by calculating temporal correlations across the brain with the time-course from a seed voxel whose spatial location was chosen from a prior finger-tapping study. It is suggested that these signal variations, temporally correlated across the brain, are of neuronal origin and correspond to functional networks in the absence of external stimuli and may reflect functionally distinct networks (Beckmann et al. 2005; Smith et al. 2009; Yeo et al. 2011; Choi et al. 2012; Seeley et al. 2007; Menon and Krishnamurthy 2019). Other studies used task-induced activity and compared to rest functional associations between various brain regions and networks. To confirm the functional connectivity pattern of brain regions, several authors use meta-analysis based on the Neurosynth data set (https://neurosynth.org). The meta-analytic connectivity modeling (MACM) measures the co-activation pattern of a seed region across a large number of neuroimaging studies. In a study Eickhoff and coworkers (Nickl-Jockschat et al. 2015) conducted an activation likelihood estimation (ALE) meta-analysis on neural correlates of face processing in autism spectrum disorder (ASD) patients. Their findings suggest a functionally and structurally disturbed network of occipital regions related to face processing, that interact with inferior frontal as well as limbic regions and may be the cause of aberrant face processing and reduced interest in faces in ASD.

Functional connectivity (FC)^11^ investigates correlations between region or source specific time series; however, as a recent theoretical review explains, it is important that research advances from FC to study effective connectivity which is the causal influence that neuronal populations exert over each other (Reid et al. 2019). Mapping between brain structure and function is not straightforward. In general, two regions may lack direct synaptic connections, but nonetheless communicate through polysynaptic pathways. Furthermore, some contexts may silence effective connectivity despite the presence of structural connectivity, as suggested by silent synapses (see review by Ovsepian 2019). In fact, using animal experiments and theoretical models, Abeles and his coworkers (Brama et al. 2015) showed synchronization among neuronal pools without common inputs and this mechanism may account for the binding activity across distributed brain areas.

Dehaene and his coworkers (Sun et al. 2016) tested the hypothesis that the variability of the cortical folding pattern in right-handed subjects is linked to the localization or the pattern of functional activations induced by hand motion or silent reading. In another elegant paper, Lupino and his coworkers (Howells et al. 2020) compare macaque lateral grasping and oculomotor networks using resting state FC, diffusion tractography and ex vivo histology based tracing. The results showed that parieto-frontal connections were best reproduced using both structural and functional connectivity techniques. Tractography showed lower sensitivity but better specificity in reproducing connections identified by tracer data. Functional connectivity analysis performed in native space had higher sensitivity but lower specificity and was better at identifying connections between intrasulcal ROIs. Anatomical–functional coupling is investigated by Bullmore and his colleagues during the sleep–wake cycle using fMRI, DTI and EEG data with graph-theoretical tools (Tagliazucchi et al. 2016). Globally, similarity between whole-brain anatomical and functional connectivity networks increased during deep sleep. Regionally, they found differential coupling: during sleep, functional connectivity of primary cortices resembled more the underlying anatomical connectivity, while they observed the opposite in associative cortices. Increased anatomical–functional similarity in sensory areas is consistent with their stereotypical, cross-modal response to the environment during sleep. In distinction, looser coupling—relative to wakeful rest—in higher order integrative cortex suggests that sleep actively disrupts default patterns of functional connectivity in regions essential for the conscious access of information and that anatomical connectivity acts as an anchor for the restoration of their functionality upon awakening.

The network interactions need to be studied across various levels (Reid et al. 2017), including at the microcircuitry, mesoscale, and macroscopic levels. While cortical circuitries can be built by a combination of basic circuitry types, including feedforward excitatory, recurrent feedback excitatory, feed-forward inhibitory, recurrent feedback inhibitory and inhibitory–inhibitory types, the actual networks that produce cognition are poorly understood (Nadasdy et al. 2006). In a few brain areas, including hippocampo-cortical networks (Buzsaki’s and Peter Somogyi and Klausberger’s groups), motor cortex (Svoboda’s group) the barrel cortex (Staiger and Petersen 2021) the basalo-cortical network (Gombkoto et al. 2021) or in some sexual behaviors organized at hypothalamic level (Karigo et al. 2021), the morphological and high-density recording tools for a complete millisecond characterization of the circuits in behaving animals may be within reach in a few years. These circuits may have evolved through evolutionary pressure but the complete micro- and macro-level blueprint of the human brain is much farther away than we thought 15 years ago, perhaps it will take 50 or100 years, although speculations to build machines to simulate consciousness, ‘the hard problem’^12^ of neuroscience and decipher with AI its organization are abundantly present in the current literature already (see Graziano 2019).

## Concluding remarks

The translation of structure into function is not a straightforward process: in addition to a weighted matrix of connections, we also need to understand the semantics by which the various brain regions converse with each other (Douglas and Martin 2007). According to Buzsaki (2019) global and local oscillations serve the syntax. While optogenetics has its own technical limitations, studies by combining viral genetic tracing, quantification of input–output connection of transmitter-specific neurons, receptors, channels, open the door for data-driven analysis of synaptic mechanisms that mediate the integrative aspects of behavior or cognition. There is increasing awareness, however, that this and other “modern” techniques have limitations: using new viral vectors without adequate controls falsely identify new connections.

Morphological studies should apply proper granularity and quantitative methods that encompass whole brain approaches. It is important to apply molecular markers that can be combined with patterned physiology (e.g., Cerminara et al. 2015); we need to include vasculature (e.g., Ji et al. 2021) and the role of glia. Neuroimaging in non-human primates needs to be combined with perturbations (Klink et al. 2021). Finally, we need to incorporate best practices from various research projects, and most of all to re-evaluate old morphological concepts, such as cortical columns, and other spatially periodic pattern of axons, cells, receptors that have gotten us almost nowhere and consider developing new views. I am confident that the new leadership of BSAF will firmly lead in this direction.

## Footnotes

1 I met first with Zilles, at the German Anatomical Society meeting, during the time I was an Assistant Professor in Würzburg from a leave of absence from Semmelweis Medical School, Budapest/Hungary in 1973/74. The Anatomical Institute in Würzburg is in the Koelliker Str, the street was named after the famous anatomist, chair of the Anatomy in the late 1890s, and a comrade in arms of Ramon y Cajal in establishing the neuron doctrine; another famous professor in Würzburg was Conrad Roentgen down the street, and the first X-ray photo was made from the hand of Koelliker.

2 I invited Dr. Pillman, a geneticist, to Stockholm in the Summer of 2006 to the Cajal Club international meeting, to the centenary of Golgi and Cajal’s Nobel Prize, an opportunity to see what a century of neuroanatomy discovery brought to neuroscience.

3 A more detailed story of the foundation of BSAF appeared in the first issue of the journal in 2007 in the Prologue (Zaborszky and Zilles 2007).

4 From business purpose Springer continued the numbering of the Anatomy and Embryology, a journal of which Zilles was Editor-in-Chief. On the other hand, Anatomy and Embryology was the continuation of Zeitschrift für Zellforschung, started in the late 1880s. BSAF started with volume 212. In 2014 we had 650 and in 2016 690 submissions.

5 Manuscripts with focus on the peripheral nervous system are not accepted for publication and the journal discontinued accepting papers related to spinal cord due to increased amount of backlog. High quality anatomy research into the mammalian central nervous system is central to BSAF, although coverage may range beyond this taxon.

6 Nicola Palomero-Gallagher, Professor, Groupleader, Institute of Neuroscience and Medicine, Jülich Forschungszentrum, and more recently an Associate Editor of the journal.

7 A brief eulogy of Karl Zilles was published in issue 225/4 (Zaborszky 2020).

8 Katrin Amunts, Professor of the C. and O. Vogt Institute for Brain Research at the University of Düsseldorf and is also Director of the Institute of Neuroscience and Medicine in INM-1 in Forschungszentrum Jülich.

9 Simon Eickhoff, Professor, Director of the Institute Brain and Behaviour (INM-7), Forschungszentrum Jülich.

10 *Nodes* are anatomically defined regions which represent the origin or termination of projections. The axons connecting pairs of nodes are the *edges* of the connectivity matrix. The *degree (or connectivity)* quantifies the number of connections from one node to the remainder of the network. *Degree distribution* gives the probability that a selected node has exactly *k* links. In random networks there are no highly connected nodes (termed also as hubs), by contrast, the degree distribution in scale-free networks approximates a power law. Distance in networks is measured with the *path length*, which tells how many edges have to be crossed to connect two nodes. As there are many alternative paths between two nodes, the *shortest path* is the path with the smallest number of links between selected nodes. The *mean path length* is a measure of the network overall navigability: represents the average between all pairs of nodes. Note the measure ‘distance’ does not reflect physical distance. C*lustering coefficient* depicts the ability of the neighbors of a node to connect with each other. Small-world networks have high clustering and small characteristic path length. Regular networks with high clustering and high path length, means that local connections prevail and that distant brain areas can only be reached via a high number of intermediate areas. In contrast, in a small-world network, with high clustering and low path lengths, distant brain areas can be reached within a small number of steps.

11 The paper by Hanson and Glymour (2010) describes some of the potential problems in extracting correct causal information from fMRI data and the perspective by Reid et al (2019) gives a theoretical background to the issue.

12 Tom Stoppard: The Hard Problem, Grove Press, New York, 2015; first produced on the Dorfman stage of the National Theatre, London, 2015; a revival of The Hard Problem produced in New York at the Newhouse Theater, 19 November 2018.
